# New challenges in structural biology: catching the complexity of dynamic nanomachines

**DOI:** 10.3389/fmolb.2014.00003

**Published:** 2014-03-27

**Authors:** Annalisa Pastore

**Affiliations:** Department of Clinical Neuroscience, King's College LondonLondon, UK

**Keywords:** in cell structural techniques, molecular machines, quantitative methods, structural biology

Why do we need *in vitro* studies? Life, of course, takes place in very complex environments. Nonetheless all what we know about fundamental topics such as DNA replication, enzyme mechanism or molecular recognition between biological molecules is the result of studies carried out on highly purified proteins. Knowing the three dimensional structure of a molecule, in particular, allows us to open a window into function (Goodsell et al., 2013). The birth of Structural Biology can be approximately dated to the ‘50ies of the last century when the structures of the DNA double helix and of myoglobin were determined at atomic resolution (Bansal, 2003; Richardson and Richardson, 2014). More than sixty years and several ten thousand structures later, we can look back and trace the developments of the structural field (Berman et al., 2013). There is no doubt that we have learned a great deal on protein structure and their function through these years. We can now, for instance, fully appreciate the exquisitely hierarchical order that rules proteins in a fantastic “legoland-like world:” globular proteins contain secondary structure elements which in turn form the basic folded units or domains which in turn form globular proteins (Caetano-Anollés et al., 2009). Nature parsimony is also testified by modular proteins which are made of the same limited number of building blocks or modules thus allowing the combinatorial construction of entirely different proteins (Nash, 2012).

Our perspective has however changed with time: Structural Biology started with people focusing on specific (globular) proteins. We then moved to protein complexes the size of which grew to reach systems as complex as the proteasome or the ribosome (Chiu et al., 2006). More recently the realization that not all proteins are compact globular entities and that many proteins are intrinsically devoid of an intrinsic ordered structure in the absence of a partner has added an additional layer of complexity to our perspective of the protein structure landscape (Stein et al., 2011). These achievements have all been possible through the development of advanced techniques which cover several ranges of resolution, starting from X-ray crystallography and fiber diffraction which remain the most established tools (Morris and Serpell, 2010; Giegé, 2013), to liquid state nuclear magnetic resonance, cryo-electron microscopy, small angle scattering (Billeter et al., 2008; Dubochet, 2012; Petoukhov and Svergun, 2013) to the more recently developed native mass spectrometry and solid state nuclear magnetic resonance techniques (Marcoux and Robinson, 2013; Opella, 2013).

Our modern perspective has also moved far from the original naïve idea of “one-protein-one-function:” moonlight proteins, that is proteins that adopt several different functions, are now considered the rule rather than the exception (Jeffery, 2009).

What next? Several different new challenges seem to be awaiting us.

**Figure 1:**
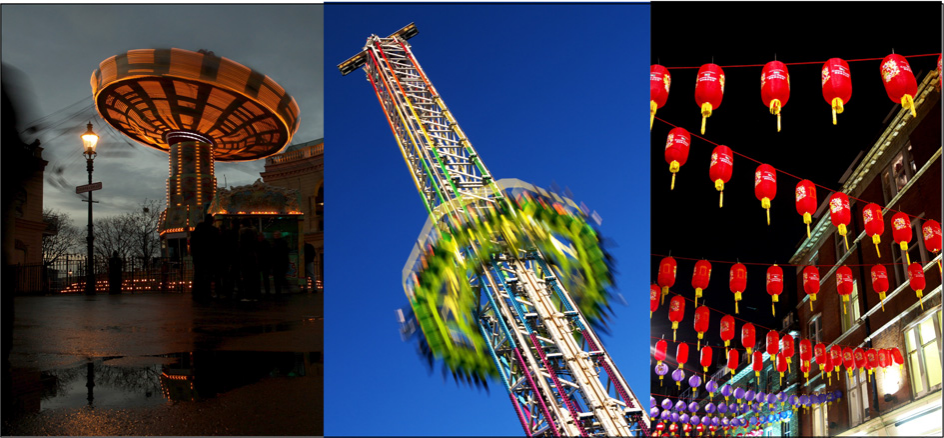
**Understanding how biological machines work is one of the main challenges of modern Biology**. Depicted here are pictures from the macroscopic world which are free interpretations of some essential nanomachines: **left**, the ATPase rotor; **center**, helicase threading along a DNA strand; **right**, the formation of a nascent protein chain on the ribosome. The pictures are courtesy of Dr. Robert Yan.

A particularly fascinating one is to understand the dynamical functioning of entire molecular machines moving beyond the description of static complexes (Figure 1). Machines are typically composed of complex networks of competing interactions which form and disassemble in a time-resolved way. As suggested by Bruce Alberts (2012), the grand challenge of the next 20–30 years will be to capture the secrets of such machines by reconstructing intricate interactomes and characterizing the various complexes formed at any specific time point and cellular location. Directly related to this problem, is the question of: how different machines talk to each other. If the same desulfurase enzyme is, for instance, involved in several different pathways, such as thiamine and biotin synthesis, tRNA modifications and molybdopterin biosynthesis (Roche et al., 2013), what determines which pathway is activated at each time point? How do the different pathways cross-talk amongst each other? Addressing these and other similar questions will require the development of entirely new technical tools and the *ad hoc* advancement of the already existing structural techniques.

The study of machines recall other related challenges: can we account in our studies for the complexity of the living cell? Traditionally, Structural Biology has been by election an *in vitro* technique in that relies on the use of highly purified proteins. This is the only way to ensure that what we observe is caused directly by the protein we want to study rather than by impurities or by mediated effects. While this concept cannot be overcome, it is however becoming increasingly important to fill the gap between biophysical studies and cellular biology: With this aim, an increasing interest is being paid to studies of molecular crowding in the attempt of designing new ways to approach structural biology in millieux as close as possible to the cellular environment (Foffi et al., 2013). As a corollary, we need to develop methodologies that may allow us to look at protein structure directly *in cell*. Amongst these techniques, *in cell* nuclear magnetic resonance is one of the most recent developments which, in principle, may allow studies of protein structure and chemical modification directly *in cell* (Ito and Selenko, 2010). Also in this field, however, we are still in the infancy of the technique. Having started with great transport, this field has more recently slowed down as it has become clear that not all proteins are amenable to these studies since the methodology seems to be some times hampered by non-specific interactions. It is thus important to find new ways that will allow us to circumvent the difficulties and tailor the technique to specific biological questions.

Another remarkable aspect that requires increasing attention is how post-translational modifications affect protein structure and modulate interactions. We have for instance seen the importance of modifications for the histone code where a few chemical groups account for the combinatorial complexity which modulates gene expression (Füllgrabe et al., 2014).

Finally, for too many years we have focused on proteins and nucleic acids, neglecting other important cellular components such as carbohydrates, lipids, and small metabolites. These studies have often been hampered by the difficulties of producing these molecules in high quantities and purity. Yet, their role in the cellular environment cannot be ignored. How do these intervene in the biological processes? How do they interact with proteins and with each other? It is about time to reconsider these questions and extend our structural studies to include these molecules.

These and many other important topics will help us to obtain an ever more accurate characterization of the mechanisms that underlie the biological processes. It can be anticipated that, while trying to find answers to these fundamental questions, we shall also achieve main developments of new, efficient approaches for the treatment of the complexity of Nature.
